# Exacerbating Pre-Existing Vulnerabilities: an Analysis of the Effects of the COVID-19 Pandemic on Human Trafficking in Sudan

**DOI:** 10.1007/s12142-023-00683-7

**Published:** 2023-05-11

**Authors:** Audrey Lumley-Sapanski, Katarina Schwarz, Ana Valverde Cano, Mohammed Abdelsalam Babiker, Maddy Crowther, Emily Death, Keith Ditcham, Abdal Rahman Eltayeb, Michael Emile Knyaston Jones, Maria Peiro Mir

**Affiliations:** 1 The Rights Lab, The University of Nottingham, Highfield House, Nottingham, NG7 2RD UK; 2 Global Partners Governance, London, UK; 3 Waging Peace, Stamford, UK; 4 Royal United Services Institute, London, UK

**Keywords:** Human trafficking, Sudan, Modern slavery, COVID-19, Pandemic, Intersecting crises, Transitional state

## Abstract

COVID-19 has caused far-reaching humanitarian challenges. Amongst the emerging impacts of the pandemic is on the dynamics of human trafficking. This paper presents findings from a multi-methods study interrogating the impacts of COVID-19 on human trafficking in Sudan—a critical source, destination, and transit country. The analysis combines a systematic evidence review, semi-structured interviews, and a focus group with survivors, conducted between January and May of 2021. We find key risks have been exacerbated, and simultaneously, critical infrastructure for identifying victims, providing support, and ensuring accountability of perpetrators has been impeded. Centrally, the co-occurrence of the pandemic and the democratic transition undercut the institutional and governance capacity, limiting the anti-trafficking response and exposing already vulnerable groups to increased risks of human trafficking. Findings point to increased vulnerabilities for individuals with one or more of the following identities: migrants, refugees, females, and informal labourers.

## Introduction

If anything, it [trafficking] has increased, less because of COVID, more because of the economic crisis it has created. You have these people coming through and you can exploit them—there’s an opportunity to extract money from them. The numbers of people had decreased quite significantly prior to COVID, and the work in the triborder area… There was a significant increase in Eritreans using air routes rather than overland, because of the reputation of Sudan and also Libya. There was less interest in taking overland routes. But with COVID and the airport closing we expected that was going to result in more people going overland [and] numbers were starting to show that at the end of last year. —*Interview, Senior Researcher ITERU, May 2021*

The United Nations Office on Drugs and Crime warned in May of 2020 that lockdown measures in place to stem the spread of COVID-19 were exposing victims of human trafficking to increased exploitation and limiting access to services. Lockdown measures were preventing victims from escaping or finding help and delaying access to legal or social services. In addition, school closures, loss of income, and reduction in support were predicted to increase risks of exploitation for vulnerable populations (UNODC 2020). Yet, analyses of COVID impacts on human trafficking have been conducted primarily on sites in the Global North or on health-related impacts, primarily mental health for victims and survivors.^1^ Other commentaries are prospective or preliminary in nature, identifying emerging trends based on anecdotal or limited evidence, or basing conclusions on likely impacts on evidence from previous crises (Coker et al 2020; Duclos and Palmer 2020; UNODC 2021b; UN Population Fund, 2020).^2^

Investigation of the relationship of the COVID-19 pandemic to human trafficking in other contexts is largely absent from the existing body of scholarly work.^3^ This is a missed opportunity, as robust analysis of the impact of COVID-19 on human trafficking in different geographies provides insights into the ways in which vulnerability is locally constituted and mitigated. In this regard, Sudan is a critical context for interrogation, not only because of the high levels of existing risk and vulnerability to human trafficking and the lack of academic interrogation but also because of the multitude of forms of exploitation along the ‘human trafficking continuum’ (Warria 2021). Sudan is a source, destination, and transit country for people experiencing or at risk of human trafficking. It ranks fourteenth of 167 countries in terms of prevalence of ‘modern slavery’ as a proportion of the population in the Global Slavery Index, with 465,000 people estimated to be living in modern slavery in 2016 (12 persons for every 1000 in the population) and many more people routinely trafficked across its borders (Walk Free 2018). The US State Department’s Trafficking in Persons report ranked Sudan ‘Tier 3’ for 15 of the past 21 years—the lowest possible rating (US Department of State 2019a, b). Recently, however, Sudan undertook a 3-year strategy to address human trafficking and was moved from the Tier 3 to the Tier 2 Watch List from 2018 to 2020, rising to Tier 2 in 2021 and 2022 (US Department of State 2021/2022). Understanding the impact of COVID-19 on human trafficking could, in turn, inform post-COVID recovery with relevance to future pandemics particularly in post-conflict and transitional states such as Sudan.

Addressing this knowledge gap, this research presents an overview of findings from a multi-scalar investigation into the impacts of COVID-19 on human trafficking in Sudan. Based on semi-structured interviews with expert stakeholders, a systematic evidence review, and a survivor focus group, this analysis finds that the pandemic has amplified vulnerabilities to human trafficking and re-trafficking. Our analysis identifies specific impacts of the pandemic on livelihood and socioeconomics, migration management, institutions and governance, and in particular the functioning of the transitional state with implications for vulnerability to trafficking. In particular, the pandemic disrupted livelihood strategies, triggering migration while systematically problematizing and illegalizing movement. This was exacerbated by the closure of protective entities, reduction in governmental presence, and diminished forms of regulation at the government and institutional level which increased risks to human trafficking while perpetration escalated. Impacts were most severe for migrants, forced migrants, informal labourers, women, and children.

## COVID-19 and Sudan

Preliminary evidence suggests that COVID-19 has had dramatic economic and social impacts in Sudan (KPMG 2020). The first positive case of COVID-19 in the country was reported on 13 March 2020. In response, the government implemented strict lockdown measures, including border closures, quarantine rules, limitations on intrastate movement, governmental closures, restrictions on public gathering, and workplace social distancing (Altayb et al. 2020). Lockdown restrictions were in place through January of 2021, and schools only reopened after a year of closure in March 2021. As of September 2021, Sudan had reported 39,550 cases and 3038 COVID-19 deaths (United Nations Office for the Coordination of Humanitarian Affairs 2021). Yet, limitations on access to equipment have constrained testing, and as a result, the actual positivity rate is likely to be much higher than the tested rate of nearly 55% (Altayb et al. 2020).

The pandemic and pandemic governance has had a ripple effect in other areas including finances. The country’s former Finance Minister Ibrahim al-Badawi estimated governmental revenue dropped 37% from previous projections in May of 2020. The IMF estimated that the Sudanese economy would shrink by 7.2% in 2020 and unemployment would increase to 25% (IMF 2020).^4^ There were differential impacts by sector and workplace conditions. In particular, a ban on public gathering instituted as a component of lockdown negatively impacted the informal economy and affected the livelihoods of millions (UN News 2020). In part as a result, seven million individuals are estimated to require humanitarian assistance Department for International Development in Sudan (n.d.), over one in five children under five is experiencing chronic wasting, and about one third are stunted by serious malnutrition (Abu-Fatima et al. 2021).

In addition to the economic realities, Sudan faces challenges to delivering an adequate response to the COVID-19 crisis. Ongoing conflicts throughout Sudan have problematised the delivery of humanitarian services and further contributed to widespread displacement and insecurity. Furthermore, Sudan is currently undergoing a political transition following the military ouster of President Omar al-Bashir—the long-time leader who took power in 1989 (BBC 2019). The reformation of the government had been managed in such a way as to balance competing interests of civilian, militia/armed groups, and the military, in addition to regional needs and varied degrees of governmental representation across geographies (Oxford Analytica 2019). The task of weaving together competing coalitions into the governing body and balancing international demands, such as turning over President Bashir, to the ICC problematized the transition. The difficult dynamics led to a military coup in October 2021 in which the interim prime minister was taken hostage and put under house arrest, as well as members of his cabinet and other civilian leaders (Beaumont 2021; Soy 2021). The subsequent resignation of transitional Prime Minister Hamdok raised legitimate concerns about the future of the democratic transition which seems to be on hold (BBC 2021). Together with ongoing conflict and degraded economic conditions, Sudan faces increasingly complex challenges to identifying priorities and resource distribution.

Until recently, there were indications of reason to be hopeful. In 2021, Sudan rose to Tier 2 in the US State Department’s Trafficking in Person’s (TIP) report ranking for the first time in the twenty-year history of TIP reporting (US Department of State 2021). Likewise in 2021, Sudan was moved from the US States Sponsors of Terrorism List (1993 up to 2020) following negotiations that included commitments to normalising relations with Israel, a settlement with the victims of the 1998 embassy bombings, and ongoing conversations around their role harbouring Osama bin Laden (Republic of the Sudan Ministry of Finance and Economic Planning, 2019). This opened the possibility for funds from the International Monetary Fund and The World Bank. However, the recent coup in Sudan led the World Bank and the USA to freeze operations and investment in Sudan, likely impeding recovery and limiting resources available for building an adequate response to COVID-19, or addressing its impacts on human trafficking (Abdelaziz 2021).

## Pre-Pandemic Sudan: Tying Specific Vulnerabilities to Common Forms of Exploitation

There is a renewed emphasis within the field of human trafficking studies on narrowing the scale of analysis and focusing on understanding place-specific risks to particular forms of exploitation (Akee et al 2010; Cho 2015; Frank 2021; Landman and Silverman 2019; Perry and McEwing 2013). Both the conditions and the form of exploitation are shaped by local geography, ‘culture’, and economy. Cultural norms around purchasing cheap goods, for instance, might contribute to supply chain demand for inexpensive labour, while elsewhere child marriage norms promote its practice and laws permit it (Gardner et al 2020). Social and community networks and their normative practices are important protective or exacerbating phenomena (ibid) and geography is a factor: artisanal gold mining is rarely an urban phenomenon. In short, explanatory factors require knowledge of the specific phenomena given the sociospatial and cultural context.

To that end, the existing literature on human trafficking through Sudan focuses on the East and Horn of Africa section of the Central Mediterranean Route (CMR), which replaced the Eastern route for migration from the Horn of Africa to Middle East (typically via Egypt, across the Sinai to Israel) (IOM 2019). The CMR is widely viewed as the most dangerous migratory route in the world.^5^ Large swaths of the migratory route require passage through the Sahara, which involves exposure to severe environmental factors often resulting in death—deaths since 2014 exceed 24,000 people (Galos et al 2017). Human-created risks are also widespread and derived largely from the dependence on smugglers to navigate the terrain; smugglers often abuse migrants, through violence or even exploitation, and can thus easily turn into traffickers or hand clients over to traffickers (Lumley-Sapanski and Schwarz 2022). Estimates from the International Organization for Migration (IOM) indicate that 73% of migrants along the CMR experience human trafficking or a form of exploitation (Galos et al 2017).

The approach to border securitization institutionalised through agreements between the European Union and external partners, like the Khartoum Process (KP) and Joint Valletta Agreement, have been critiqued as contributing factors to the exploitation and abuse of migrants and refugees (Reitano 2017; Tubiana et al. 2018; Davitti 2018). As individuals seek to avoid border enforcement, they are pushed onto less visible, less regulated migratory routes with more risks of victimisation (Lumley-Sapanski and Schwarz 2022). Additionally, the KP prioritises cooperative solutions between African and European partners to fight “irregular migration, migrant smuggling, and trafficking in human beings” (Tewolde-Berhan et al. 2017) by training “law enforcement and judicial authorities” in new methods of investigation and assistance (Tewolde-Berhan et al 2017, pp. 12–13). Yet, military and border officers within Sudan and Eritrea are known to participate in trafficking of human beings problematizing this partnership and potentially increasing risks to migrating populations (Plaut 2017).

Within this context, individual vulnerability to trafficking follows common social fault lines, and vulnerabilities are frequently multiple and intersectional. In general, impacts of contextually specific risk factors and multi-scalar vulnerabilities occurring across multiple scales contribute to increased vulnerability for particular populations (Byrne et al 2021; Cho et al. 2013). The International Organization for Migration’s Determinants of Migrant Vulnerability (DoMV) identifies determinants of vulnerability to exploitation, abuse, and violence broadly including trafficking. The DoMV points to vulnerabilities across four scales: individual, household, community, and structural and understands vulnerabilities as cross-cutting and interrelated (International Organization for Migration 2019).

How is trafficking evidenced in Sudan and what are common vulnerabilities? Exploitation type varies given individual attributes, and, communal and place-based risk factors (e.g. prevalence of child marriage). Most commonly refugees and migrants are kidnapped and tortured to extract money from their diasporic connections. Women are often the victims of sexual and gender-based violence and torture (UNHCR 2020). Refugees, and particularly Eritreans, are abducted for sexual or labour exploitation and taken to Libya (UNHCR 2020). Upon reaching Libya, refugees continue to experience torture, extortion, and forced exploitation (sexual or labour) up to and including death (Kuschminder and Triandafyllidou, 2020; UNHCR, 2021). It is highly profitable: prior scholarship has estimated that approximately 30,000 Eritreans were trafficked or smuggled through the Sinai (via Eastern Sudan) between 2009 and 2013, for a total net benefit of 600 million USD (van Reisen and Estefanos 2017).

Refugees and asylum seekers are particularly at risk. Sudan hosts millions of refugees, but their rights are restricted through a strict encampment policy (Hovil and Oette 2017; Lumley-Sapanski et al., 2021). Refugees are denied the right to free movement within the country, most lack access to education beyond primary school, and refugees cannot work legally. This restricts their income options, leaving them dependent on insufficient rations. Furthermore, widespread corruption and harassment from police and militia entities create a climate of fear driving onward migration and the use of a smuggler to evade said security forces (Ibid). Former refugees also report being kidnapped and abducted from refugee camps and trafficked or sold to traffickers by border police ostensibly responsible for taking them to camps (Interviewee N; Lumley-Sapanski, in press).

More broadly, children and women with other vulnerabilities (e.g. migratory status) are more likely to experience exploitation or trafficking. Women in poverty, female migrants and IDPs working as domestic workers, and forced migrants are common victims of human trafficking and exploitation (Lumley-Sapanski and Schwarz 2022). Sudanese traffickers often exploit migrant women and IDPs in domestic worker roles within private homes in Khartoum and other urban centres (US Department of State 2020). Migrant women, especially Ethiopian women, are forced into commercial sex in Khartoum by criminal networks who manipulate debts or through other forms of coercion (US Department of State, 2021, 2020, 2019a, b).

Children are at heightened risk in conflict zones of which there are several, in urban spaces with concentrated poverty, and in artisanal mining areas. They may be forcibly recruited by governmental and non-governmental armed groups in conflict zones; unaccompanied minors who are also IDPs are frequent targets due to their lack of protection. Traffickers also exploit children who are homeless in Khartoum through forced labour for begging, and in industries such as in brickmaking, for the purposes of gold mining, collecting medical waste, street vending, agriculture, and in the sex industry (US Department of State, 2021, 2020, 2019a, b). Additionally, child marriage is practiced and more prevalent in times of economic crisis; the legal age of marriage was 10 until 2020, and 38% of married women were married before 18 (Chr Michelsen Institute 2017).

While gender, endemic poverty, and migratory status shape vulnerabilities within Sudan, lack of governance and governmental participation in trafficking influences the prevalence of human trafficking. In some instances, they have been accused of participating, while elsewhere, they are blamed for profiting from and permitting the practices of human trafficking (Lumley-Sapanski forthcoming; Lumley-Sapanski et al. 2021). This underscores the need for analysis of human trafficking—and COVIDs impact—from a multi-scalar perspective that accounts for structural factors in addition to individual, community, and household attributes.

## Methods and Data

To understand the impacts of the pandemic on vulnerability, practices, and prevalence of human trafficking in Sudan, required an in-depth analysis of ‘what was known’ in these areas pre-pandemic and a subsequent assessment of the impact of COVID-19. To this end, this project combined three layers of qualitative research and analysis using primary and secondary data sources: a systematic review of existing and emerging evidence; in-depth semi-structured interviews with key informants to understand the emerging impacts of the pandemic; and findings from a focus group held with Sudanese survivors in the UK.^6^ This approach provided triangulation of data and analysis, as well as validation by people with lived experiences of trafficking.

The systematic review was used to map the current state of knowledge, compiling and analysing academic and grey literature, as well as relevant legal, regulatory, and policy standards. Due to the recent and rapidly changing nature of the topic (COVID-19), newspaper articles and other forms of popular media were also compiled and analysed. A term harvesting template and search tracking sheet were used to record searches, results, and reasons for exclusion throughout the review. A total of 94 pieces of evidence were selected for inclusion and subsequently thematically coded in NVIVO. Data collection was conducted from 25 January to 02 February 2021. Evidence published after this date may therefore not have been considered in the review. Evidence reviewed was limited to that published in English from 2018 to 2021 and accessible online.

Interviews provided in-depth insights into the effect of the pandemic on human trafficking in Sudan as it was unfolding. These interviews were particularly important for understanding the response to the pandemic by institutions, the empirical impacts of the pandemic on institutional capacity and functionality, and the perceptions of the pandemic’s impacts on human trafficking by institutional actors responsible for assessing and monitoring these impacts. Twenty-two interviews were conducted with experts working in anti-trafficking or modern slavery governance, abolition, criminal justice and legal systems, or humanitarian work. These included amongst others: United Nations High Commissioner for Refugees, Regional Operational Centre in support of the Khartoum Process and AU-Horn of Africa Initiative, Better Migration Management, GIZ, National Committee for Countering Human Trafficking, Danish Refugee Council, United Nations Children’s Emergency Fund, the UK Foreign, Commonwealth & Development Office, Global Partners Governance, Human Rights Watch, Sudan Social Development Organization, and Waging Peace. A combination of purposive and snowball sampling was used to identify respondents. Interviews lasted 45 min to 2 h and were conducted using zoom or Microsoft Access (due to the pandemic) in both English and Arabic. Interviews were recorded and subsequently transcribed and translated where necessary, with three exceptions (where interview notes were analysed). Thematic analysis of interviews was conducted in NVivo. Selected insights from interviews are included here, reported using organisational affiliation for anonymity.

A two hour focus group was held with survivors in the UK to discuss outputs from phase one and two. This discussion was used to ensure the voice of affected communities were incorporated into the data collection and research analysis phases. Participants were presented with a brief list of findings and recommendations along five key themes: domestic worker vulnerabilities, displacement camps as recruitment sites, debt bondage, diaspora communities, and compounding structural factors. Eight survivor participants attended the focus group discussion. Responses were invited in both English and Arabic, with simultaneous interpretation provided. Survivors were also invited to share an additional reflection with the focus group organisers, with two survivors submitting written supplementary insights. Survivor reflections on emerging findings and recommendations from the evidence review were used to shape analysis and, ultimately, the prioritisation of recommendations. Their insights are incorporated into the recommendations and discussion below. This approach positioned survivors not as the subjects of research, but as co-creators of research outputs. Wraparound support for survivor participants was provided by organisers.

### Analysis

Analysis is divided into the pandemics impact in four key areas: livelihood and socioeconomics, migration management, institutions and governance, and the transitional state. We observe the increased vulnerability of already marginalised groups, driven by the closure of protective entities, diminished forms of regulation, and reductions in resources driving migration while problematizing access to safe migration.

#### Migrants, Migration, and Migratory Status

Last year as a result of the pandemic…the smuggling and trafficking continued in normal terms but the closure of the border, it led the smugglers and traffickers to use different routes, not actively used in past. We really have seen some changes and trends in arrival and departure…many people who lost their jobs in the neighbouring countries or the visas were expired had no choice and had to return. —*Interview, UNHCR Officer, April 2021*

As described by the UNHCR employee above, coronavirus and the associated lockdown restrictions had implications for governance over and enforcement of borders, movement, and gathering. The restrictions presented specific challenges for potential migrants and migrating populations, simultaneously increasing both migration ambition and problematizing mobility. Irregularized migration was more common due to closed borders with implications for the safety of migrants and risks to exploitation and human trafficking.

First, the initial governmental response to COVID increased certain forms of migration including ‘forced’ irregular migration with inherent risks. Primarily, socioeconomic deterioration exacerbated by pandemic restrictions on business operations and public gathering (e.g. the closure of tea stalls and markets) drove migration as a coping strategy. People with limited social welfare options, lacking support infrastructure, or other livelihood prospects were driven to consider onward migration. As the below interviewee implies, the limitations on legal migratory routes drove irregular migration with attendant increased risks to trafficking:

The pandemic has had a huge impact on human trafficking. The economic downturn caused by the lockdown has impacted low-skilled and ‘blue-collar’ jobs that made people resort to alternative means to generate revenue, including migration. This increased their likelihood of falling victim to human trafficking. —*Interview, Core Member, National Committee to Counter Trafficking, April 2021*

Other examples of pandemic-induced migration with increased risks to trafficking include the management of expelled Sudanese immigrants from neighbouring states (UNOCHA 2021). The lack of governmental capacity to collect, protect, and reintegrate expelled Sudanese persons created an opportunity for traffickers and smugglers. This was described by one UNHCR employee:

There was a huge influx of expelled migrants…the government started receiving the cases [expelled] from Libya, in northern state…At one point they refused to receive these stranded Sudanese…very remote area, no facilities and [the expelled] Sudanese had no way to leave that area to reach their homes. They didn’t have a lot of resources. It takes three days by road to reach this triangle… They managed two or three convoys last year, but the last convoy, last December out of 140, 36 were positive, and when the government came to, they prevented the IOM from bringing the Sudanese back. —*Interview, UNHCR Representative, April 2021*

Expelled and with insufficient resources to return home, migrants were made reliant on smugglers—the only transportation option for most and susceptible to exploitation. This underscores the combined impact of the state’s response to coronavirus and approach to managing migration on individual vulnerabilities. Chiefly by failing to provide an adequate response—either repatriation services or sequestration of positive cases—and limiting mobility options or work of multinationals, the Sudanese state made mandatory the use of smuggling and trafficking operations.

Second, the closed borders made it more difficult and more precarious to migrate in general. Irregular migrants in Sudan were made more visible as ‘out of place’ by the proliferation of laws around migration and limitations on access to public space. This increased migrant dependency on the use of smugglers or traffickers to avoid detection. Traffickers and smugglers took advantage of this power dynamic. They held the increased risks of arrest and deportation associated with their irregular status over migrants. A Child Protection Officer with UNICEF described the impacts of the coronavirus on the relationship between the smuggler and the smuggled in the following way: ‘Before COVID, some of the people in trafficking…they escape from those who have trafficked them. While during COVID-19, everything is closed, without any movement. The persons who are in trafficking [victim], he doesn’t try to take his own way. He’s fully under the detention of the trafficker’ (Interview, Child Protection Officer, UNICEF April 2021). In short, whereas in ‘normal’ times, migrants could use regular migration routes, the pandemic restrictions limited transportation options—something traffickers took advantage of to maintain control and prevent escape. This unduly impacted irregular migrants who were conditioned not to seek help from authorities due to their status.

The economic impacts caused by pandemic restrictions also impacted the business and costs of migration. The fees charged by smugglers increased in turn creating conditions for increased exploitation. The securitisation of the border and the attendant increases in risk to the trafficker drove these costs. A researcher with the Forum for Social Studies in Addis Ababa captured the impacts: ‘The major factors are when the governments on both sides, tighten up security and clamp down on smugglers, the price significantly increases’ (Interview, Researcher, Forum for Social Studies, Ethiopia, April 2021). In some cases, these were financial costs (van Moorsel 2021), and increases were cited of up to three or four times the normal charge. Interviewees also identified increased risks to exploitation and sexual exploitation that resulted from the ‘trickle down’ of costs, to abuses as a form of compensation (Interview, Child Protection Officer, UNICEF, April 2021).

Third, migration routes themselves were made more dangerous. COVID-19 closed formal borders, common irregular routes, and stopping places as much due to stigma as to policing. Places (towns, cities, etc.) which were known refuelling stations along the routes barred entry, driving smugglers and traffickers into more dangerous terrain with less visible governance. As a former Sudanese governmental cabinet member stated, ‘In the past, people en route to Europe through Sudan would pass through Khartoum, where the large Eritrean/Ethiopian population offered a support system on their journey. The pandemic meant that potential victims cannot stop through these places’ (Interview, former Sudanese Cabinet Member, April 2021). This in many cases elongated the journey or forced the use of more dangerous invisibilised routes. Migrants faced increased environmental risks to starvation and dehydration as well as the likelihood of victimisation by militias and rogue actors known to kidnap and sell victims (e.g. in Southern Libya) as a result. COVID impacted the ability of communities to welcome travellers, in some cases closing way stations and driving longer journeys.

#### Institutional and Governance Gaps

Lack of institutional access and the failures of central institutions are cross-cutting factors that influence risks to human trafficking across Sudan with differences by region. Broadly, this concerns institutions such as reproductive health centres and schools, which were initially shuttered by the pandemic restrictions. Their closure removed for many what was the only form of external institutional engagement. These institutions had provided important wellness checks, resources, and were spatially dispersed and accessible. The closure of these monitoring entities contributed to the invisibilisation of perpetration and reduced opportunities for victim identification. This had noted implications for women and children at risk of sexual exploitation and trafficking (UN Population Fund, 2020).

Non-governmental institutional closures (e.g. NGO entities) were widespread impacting victims of trafficking or potential victims of trafficking through reduced access to social welfare or other supportive services (Save the Children 2020). Significantly, COVID closures reduced the number of safehouses in Sudan. This occurred for two reasons. First, external donors experienced financial distress caused by the pandemic. This led to the closure of safe houses according to a former employee of the Regional Operations Centre in Khartoum, an organisation aimed at eradicating criminal networks: ‘There was a couple of safe houses, but none are funded by the government…They were struggling with donations from various member states, but then during COVID that money dried up’ (Interview, Former Trainer, Regional Operations Centre Khartoum, April 2021). In addition, of those which remained open, failure to comply with COVID restrictions from the Ministry of Health led to the closure of at least one safe house. Residents were asked to leave immediately presenting a challenge for their ongoing safety: ‘The situation in the houses did not comply with *COVID* 19 instructions and the Ministry of Health of the Sudanese government already by the law, closed the houses’ (Interview, UNICEF Case Officer, April 2021). In doing so*,* COVID-19 closures eliminated spaces where trafficking victims could seek protection from perpetrators and forced current residents onto the streets. This created risks for women without migratory status without other options for safe houses.

The capacity of other non-profit entities to deliver services was similarly affected. For UNHCR, a helpline replaced in-person services. A UNHCR representative underscored that a helpline could not adequately replace services to vulnerable persons and reduced the number of persons reached or served: ‘We put in place the helpline and anyone who wants to seek support from UNHCR and partners, they could seek us out. And of course, the IOM I believe in Khartoum and in the east, I think it was also affected. Pandemic affected vulnerable communities, VOTs [victims of trafficking] and others, to receive timely services’ (Interview, UNHCR Representative, April 2021). Similarly, International Planned Parenthood Foundation was closed. This left some areas—like IDP camps—with no access to healthcare or reproductive services; IPPF is a key place for identification of sexual exploitation and trafficking, and without it, practitioners raised the concern that VOT were going unidentified (International Planned Parenthood Federation 2021).

Finally, in addition to NGO closures, the lack of formal governance entities during the pandemic compounded vulnerability reducing the identification of survivors or persecution of perpetrators. Police operations were halted, the judiciary closed, and the government reduced to 50% operational capacity. The closure of the judicial system to in-person court cases was as a further factor increasing perpetration: without a system in place to process criminal cases, there was nobody enforcing anti-trafficking law leading to a reduction in prosecution of cases. Additionally, the police were directed not to arrest and bring in perpetrators, due to a lack of separate cells and concerns over spreading COVID within the prison system and to the police. This reduced identification of perpetrators or victims. The combination of the closure of the courts system, the police directive to end routine operations, and a 50% reduction in government staff contributed to the perception within interviewees that traffickers were being permitted to act with impunity.

#### Livelihood and Socioeconomics

‘The organization is providing them with masks, but they’re looking at essentials…there is a shortage of food.’—*Focus Group Participant, March 2021*

Poverty and socioeconomic degradation are previously identified factors which decrease access to resources and can increase vulnerabilities to exploitative targeting (UNODC 2021a). The pandemic exacerbated poverty leading to extreme deprivation, as the interviewee acknowledges. Given this, it is unsurprising that the data considered in this study indicates that lockdown restrictions contributed to increases in poverty and, with it, human trafficking. The impact was multidimensional. Economic deterioration was observed to disproportionately impact individuals whose incomes were from informal sources or non-regulated industries, labourers in invisibilised industries like domestic workers, and refugees and asylum seekers in camps with limited employment options or access to few economic resources.

Individuals who were self-employed or worked in the informal labour market were more likely to suffer job loss due to pandemic restrictions (for instance, migrant tea stalls at the public market). The extended loss of income was unsustainable for informal workers, a group in which marginalised populations including women, children, and displaced groups tend to be over-represented. A former criminal prosecutor in Khartoum described this impact, ‘During the lockdown, migrant communities have been deeply affected by the safety measures, because…most worked in the informal sector, and they were not allowed to render activities, many depend on their daily income… I am sure this has led to more exploitation’ (Interview, Former Prosecutor (Khartoum), April 2021).

Focus group participants validated this concern. Specifically, during the focus group, they spoke of exploitation by police officers who took advantage of the economic vulnerability of informal workers. In this case, women running tea stalls were rendered unable to earn income by the lockdown measures. When they violated the rules, corrupt police were accused of exploiting women sexually as a substitute for fees:

The tea ladies, they are mostly from Ethiopia or Eritrea. If the police find them, they have to pay a tax or something--sexual exploitation. It’s mostly about the police because the police themselves, they’re not being watched by the government. This is the result of this chaos. —*Focus Group participant, M*

Informal workers had fewer saved resources, leaving them with a little safety net when public gathering restrictions went into place and many informal jobs disappeared. In some cases, this increased migration aspirations, again problematised by closed borders. In others, interviewees expressed concern about reliance on negative coping strategies like child marriage (a practice used in Sudan during economic hardship involving the exchange of dowry) (Interview, Global Partners Governance, April 2021).

These impacts were more severe for groups facing additional mobility and labour restrictions. The Sudanese encampment policy limits employment options for refugees in camps rendering them largely dependent on aid and informal work to survive. Without the right to work in the formal economy, refugees and other humanitarian groups rely on the informal economy to meet their needs. These groups were more likely to lose their livelihoods and income as a result of their clustering in this sector (Van Moorsel 2021). With fewer resources to fall back on, they became vulnerable to targeting by exploitative traffickers. Another UNHCR officer described the camp conditions and their implications: ‘COVID-19 which restricts freedom of movement and work…that also…contribute[s] to the onward movement of refugees and asylum seekers’ (Interview, UNHCR Officer, April 2021).

Traffickers target these spaces, preying on economic vulnerability. The former ROCK employee referred to refugee camps as ‘honey pots’ in interviews: networks connected to traffickers operate in camps via local contacts to coerce migrants into joining them in onward migration—a relationship that frequently turns into human trafficking with kidnapping and debt bondage at latter stages. The lack of security within camps was reiterated by former camp residents in the focus group. When asked about the needs in the camps, these former residents emphasised the need to address risks to exploitation, violence, and death in camps over funding education or training: ‘Security is the main concern. When you have kids, you’re not thinking about education, you’re thinking about how your kids learn to protect themselves. They’re not getting it from the government’ (Focus Group N). Encampment policies combined with COVID-induced restrictions on the ability to work produced vulnerability to targeting by traffickers aware of the untenable economic conditions. The state or camp management was unable to provide security to refugees. An officer of the Norwegian Refugee Council responsible for camp management described the situation:

Many refugees don’t actually have a formal right to work. The fact that there is an encampment policy for hundreds of thousands of refugees who could be there for two or three generations by now and there are still no proper legal ways to generate an income…it increases vulnerability to modern slavery, forced labour, risks of trafficking and onward movement. —*Interview, Norwegian Refugee Council, May 2021*.

The pandemic also disproportionately impacted the vulnerabilities of women in the domestic worker industry increasing their risks to exploitation. Prior literature has shown that individuals employed in occupations which are more invisible or less regulated (such as agricultural workers and informal labourers) are more likely to experience exploitation (UNODC 2020). This is particularly true in Khartoum where most domestic workers are concentrated—there they live with their employers and lack community support. Risks arise from the isolated working conditions and the lack of cultural and legal knowledge, including labour laws:

There is a very big community of domestic workers, migrant domestic workers, from mostly Ethiopia and Eritrea, and there are a lot of domestic workers from Sudan. They are not IDPs, but they are disconnected from their regions—the northern areas, the Nuba mountains, [the area] bordering South Sudan. As a result of this, [and] the work conditions…not really connected to the community, living and working in Sudanese households, they were already… quite vulnerable in terms of human exploitation. —*Interview, Former Prosecutor (Khartoum), April 2021*

Focus group participants described the situation as widespread, one in which women from these communities were not paid minimum wage and isolated, cut off from their communities. Several said the abuse that domestic workers described was ‘unbelievable’ until they themselves were trafficked to Libya and had an experience of exploitation. Stakeholders reported increases in women being coerced or misled into trafficking situations for the promise of domestic work by economic precarity. Lockdown restrictions increased isolation and, by eliminating access to public space, gave more control to employers. This substantially contributed to exploitation and violence of and directed towards domestic workers.^7^

#### The Transitional State

‘At the particular moment for the country, in the moment that Sudan should have expected support directly and aid and support to move beyond Bashir there wasn’t that much support because all these countries—the gulf included—had turned inwards to worry about their own concerns.’—*Interview, International Crisis Group, May 2021*

This study set out to evaluate the relationship of COVID-19 to trafficking focusing on Sudan. Its identity as a place of transition emerges as a significant factor in determining the government’s efficacy and access to resources. First, while core staff were in place, with an ambitious plan to combat human trafficking, the bureaucratic processes and accompanying civil servants were described by interviewers as ‘in disarray’ due to the reorganisation implemented during the transition (beginning in 2019). The Dismantling Committee had recommended the ouster of many existing governmental personnel. Though widely seen as positive, this led to the dismissal of a great number of line staff in the Sudanese government. Their purge represented a huge loss of knowledge, with implications for the identification of victims and prosecution of perpetrators, ‘There’s a huge lack of institutional memory across the board as far as government is concerned. There are challenges across all these areas’ (Interview, Consultant, Global Partners Governance, May 2021).

The collective institutional memory loss and gaps in staffing of civil service positions impeded trafficking policing and prosecution. Focus group participants were acutely aware that the Transitional Government was perceived to not have the same information gathering capacity or central knowledge as the previous government, ‘The transitional government, they don’t have the experience, besides the experience, they’re not aware of everything that was going on. [The previous government] was aware of everything that was coming in and going out. They had a lot of people all over the place’ (Focus Group Participant, N, March 2021). The transitional nature of the government and the significant overturn in employees meant that, while there was legislation in place, there was little bureaucracy to actualise policy and intelligence gathering was reduced. When COVID-19 hit, it further interrupted momentum by reducing capacity and limiting resources available from which to rebuild. This reduced victim identification and perpetrator arrests.

An additional concern was the impact of the fiscal crisis induced by the pandemic and transitional government on the existing corruption of security and police forces. Corruption within the security forces and police was acknowledged to exist prior to the transition, but both stakeholders and focus group participants were concerned that it would increase as a result of the pandemic’s impact on the economy.^8^ With insufficient incomes combined with inflation, the financial situation of security forces and police was deteriorating and with it social safety, ‘The trafficking police they don’t have enough income. They would want something out of that person they arrested, any kind of money or things…they don’t care about, you know, the trafficking or the policing,’ (Focus Group Participant A). This was reiterated by stakeholders working with the police who explained that due to insufficient incomes, police targeted marginalised communities—in this case, he specifically referenced informal hairdressers and tea stalls run by migrant populations: ‘They’re targeted by the local police cause the police got no money. So, there is corruption. People are being approached being told to pay up.’ (Interviewee, The ROCK). In effect, the police were ‘shaking’ down migrants for bribes. Interviewees expressed a lack of institutional authority and institutional trust exacerbated by the pandemic and corruption which undercut the effectiveness of anti-trafficking interventions.

Finally, the evolving nature of the government was also seen as precarious and unstable. External international partners were hesitant to invest in or to fund the new government without clarity about its longevity or stability. Furthermore, the composition of the various parties to effect buy-in—centrally including the Rapid Support Forces (the former Janjaweed Militia)—was problematic for external funders aware of the participation of RSF fighters in ethnic cleansing in Darfur. Consequently, significant economic support from international funders that would likely otherwise have been expected, given the positive response to the governmental transition, did not arrive. This, and the reductions in available funding for anti-trafficking work resulting from the pandemic, limited resources available for funding this work within the country. This had serious implications for governance, during the critical transition period given the reliance on external support.

## Conclusion

This paper presents findings from a multi-scalar investigation into the impacts of COVID-19 on human trafficking in the transitional context of Sudan. Findings identify a significant impact of the pandemic and related lockdown restrictions on vulnerabilities to human trafficking in four areas: livelihood and socioeconomics, migration and migratory status, institutions and governance, and the overarching role of the transitional state. Analysis indicates that the pandemic disrupted the capacity of criminal justice, governance, and humanitarian organisations to provide services to survivors or to prosecute perpetrators. These disruptions further exacerbated the effects of the pandemic and COVID-19 mitigation strategies on vulnerable groups. Those with intersecting vulnerabilities—namely migrants, informal and domestic workers, women, and refugees—were the most impacted.

The confluence of these factors highlights how vulnerability and risk to human trafficking occur and intersect across multiple scales within Sudan. In this case, rigid border enforcement encouraged irregular migration, and a lack of functioning criminal and judicial institutions, safe houses, and border forces decreased the identification of cases of trafficking. Migratory status was weaponised by traffickers who benefited by leveraging their power over trafficked persons. Findings also draw attention to the role of the pandemic in undercutting the capacity of institutions at multiple scales by eliminating sources of funding, ‘distracting’ resources, and shuttering dispersed entities positioned to provide place-based interventions and care. The pandemic lockdown restrictions exacerbated extreme economic precarity and vulnerability to predation for many but, in particular, those with cooccurring pre-existing vulnerability.

Furthermore, the research provides novel insights into the significance of the transitional context, on the pandemic’s impacts on human trafficking. Specifically, we identify how the transitional nature of the government affected capacity and external support. First, the perception of instability within the government—which proved true with time—reduced the willingness of external partners to support the government financially. In addition, the inclusion of the RSF fighters in the transitional government—an attempt to build a cohesive transitional government—led international partners to resist funding border patrol and other specific ‘security’ needs for fear of supporting human rights violators.

This reality demonstrates the problems for a post-conflict transitional government building an internal coalition of disparate groups while engaging international partners. Differing institutional priorities can hinder access to resources or cause gaps in support (Ricks and Doner 2021). In this case, individual non-profits and non-governmental actors withdrew dollars from Sudanese projects due to the pandemic, which had further ramifications for a state struggling to secure external development or aid. This was particularly problematic for the Sudanese, ineligible for debt restructuring or International Monetary Fund dollars, causing them to rely on evaporating aid (BBC 2020). The pandemic reduced the capacity of the state to develop and implement a coherent response. Second, the near complete staff turnover in the civil service caused by the transition resulted in significant gaps in services and anti-trafficking knowledge. The pandemic further weakened the government and civil service capacity by reducing staffing to 50%. Reduction in capacity, knowledge, and income resulting from the combined impacts of the pandemic and the transitional state impaired the response to trafficking and governance more broadly.

Human trafficking dynamics in, through, and from Sudan are heavily influenced by regional and global events and trends. The COVID-19 pandemic is one such event, but an event which occurred in conjunction with other pre-existing local and regional crises including the autocratic government in Eritrea and the Tigrayan conflict in Ethiopia. Limitations on access to international development support in combination with the secession of South Sudan have severely crippled the economy. These crises continue to impact the Sudanese context and stretch already limited resources. As one respondent noted, the pandemic ‘is just one more piece’ in an already ‘deadly’ situation. Thus, rather than creating new risks and vulnerabilities, the pandemic predominantly exacerbated pre-existing individual, family, community, and structural dynamics which drive trafficking and exploitation in Sudan and the wider region.

Consequently, the primary impacts of the pandemic do not radically alter the manifestations of human trafficking in Sudan but intersect with pre-existing vulnerabilities to exacerbate risk and push increasing numbers of people into precarity. These have been particularly grave for migrants because of the approach to migration governance taken within the region. Encampment and border securitization policies have driven migration aspirations while limiting opportunities for seeking safety, creating chokepoints that human traffickers exploit. Efforts to address the impacts of the pandemic in Sudan in the long term must therefore look beyond the virus itself, considering the diversity of intersecting factors across scales that existed prior to the spread of COVID-19. This recommendation aligns with calls from the United Nations for the implementation of a human rights-based approach in post-pandemic settings. At its heart, a human rights-based approach is ‘a conceptual framework that is normatively based on international human rights standards and that is operationally directed at promoting and protecting human rights’ including those of trafficking survivors (UN Women 2020). Embedding the protection of human rights into governance frameworks, building collaborations with human rights agencies, challenging social norms, and continuing and extending social services (ibid) are initial first steps in redressing inequalities exacerbated by the pandemic response.

## Footnotes

1 See for example: Todres Diaz (2021) COVID-19 and Human Trafficking-The Amplified Impact on Vulnerable Populations’, JAMA Pediatrics 175, 2: 123–24; Armitage and Nellums (2020) COVID-19 and the consequences of isolating the elderly. The Lancet Public Health, 5, 5.

2 Duclos and Palmer (2020) Background Paper: Covid-19 and Forced Displacement in the Middle East and East Africa, Briefing, Social Sciences in Humanitarian Action Platform.; UNFPA (22 March 2020) Sudan GBV Sub Sector Guidance on Covid-19, 1st Bulletin, Retrieved 30 April 2021, from https://reliefweb.int/report/sudan/sudan-gbv-sub-sector-guidance-covid-19-1st-bulletin-22nd-march-2020

3 For exceptions see Warria (2021) The Human Trafficking Continuum and COVID19: Rights, Risks and Recommended Protective Actions*.* Journal of Social Development in Africa. Niezna et al. (2021) Underlying Conditions: The Increased Vulnerability of Migrant Workers Under COVID-19 in Israel, Journal of Modern Slavery: A Multidisciplinary Exploration of Human Trafficking Solutions, 6, 2: 133.

4 The June update to the report noted that the economic impact of the pandemic had, in fact, been worse than predicted, with recovery then considered to be more gradual than previously forecast. International Monetary Fund, ‘World Economic Outlook: The Great Lockdown,’ (April 2020). Available from https://www.imf.org/-/media/Files/Publications/WEO/2020/April/English/text.ashx

5 This figure is based on estimates from the IOM Missing Migrants project (https://missingmigrants.iom.int/). Deaths and violence against migrants fluctuate month over month and by region. While the Mediterranean has had the largest number of missing migrants since 2014 by a significant amount, there are emerging areas of growing violence such as the Darien Gap in Panama which are an area for concern. The Americas in the same period have the largest percentile growth in missing migrants.

6 Prior to initiating research, the proposed research was presented to the ethics committee (EC) housed within the (*) and received approval in January of 2021. Signed and informed consent was sought from stakeholder participants (form approved by EC). Verbal consent was sought from focus group participants following review of the research information sheet (which was approved by the EC. Data protection measures were put into place, compliant with university policy and GDPR.

7 See a further discussion of the phenomenon observed in Middle Eastern countries by Begum (2020) Domestic Workers in Middle East Risk Abuse Amid COVID-19 Crisis: Governments have a responsibility to protect foreign domestic workers, Human Rights watch. Retrieved from https://www.hrw.org/news/2020/04/06/domestic-workers-middle-east-risk-abuse-amid-covid-19-crisis

8 US Department of State Trafficking in Persons reports from 2022 note that incidents of sexual exploitation of refugees have been reported in refugee camps in Sudan for the past three years (US Department of State 2022).
